# First report of a stunt nematode *Tylenchorhynchus zeae* on corn in Gansu Province, China

**DOI:** 10.21307/jofnem-2020-009

**Published:** 2020-03-17

**Authors:** Zhi Peng Xu, Hui Xia Li, Yong Gang Liu, Bao Cang Ren, Chun Hui Ni, Jin Hui Ma

**Affiliations:** 1 College of Plant Protection, Gansu Agricultural University/Biocontrol Engineering Laboratory of Crop Diseases and Pests of Gansu Province, Lanzhou 730070, Gansu Province, China; 2 Institute of Plant Protection, Gansu Academy of Agricultural Sciences, Lanzhou 730070, Gansu Province, China; 3 Gansu Academy of Agri-engineering Technology, Wuwei 733006, Gansu Province, China

**Keywords:** Corn, First report, Northwest China, Stunt nematode, *Tylenchorhynchus zeae*

## Abstract

During a survey of plant parasitic nematodes in 2019, at Gansu Province, China, the stunt nematode *Tylenchorhynchus zeae* was found parasitizing corn seedlings. Females, males and juveniles of *T. zeae* were observed on soil and roots samples of corn after processing and extraction. This population of stunt nematodes was identified based on morphological and by sequencing the ITS1 region of rDNA and D2/D3 fragments of the 28 S rRNA. The ITS1 and the D2/D3 sequences of this population, shared 99.00 to 99.05% and 99.43 to 99.73% of similarity with sequences corresponding to *T. zeae* in GenBank, respectively. This is the first report of *T. zeae* infecting corn in Gansu Province, northwest China.

Stunt nematodes (*Tylenchorhynchus* spp.) are important plant parasitic nematodes that feed on a wide range of economic hosts. A survey of plant parasitic nematodes was performed in corn fields in May 2019, finding corn seedlings with symptoms like yellow leaves, dwarf plants and death plants in the field (N 37°07′36″, E 102°54′08″) of Baiyin City, Gansu Province, China. The rhizosphere soil samples and roots of diseased plants were collected. Each soil sample was about 1 kg consisted of over 20 corers using a soil auger of 20 cm in length and 20 mm diameter. Nematodes were isolated using modified Baermann technique (Hooper, 1990). On average, 5 females and 13 juveniles were obtained from 100 g soil, and 18 males and 2 females were extracted from 50 g of roots. Females bodies were cylindrical, slightly ventrally arcuated after fixation by gentle heat. Cephalic region with annules, and significant curling at the connection with body contour. Stylet with anteriorly flattened knobs, and median esophageal bulbs well developed. The vulva was located in the center of the body, and reproductive system didelphic amphidelphic. Tail conoid, with obtuse smooth terminus and phasmids almost located in the middle of the tails. Males bodies were similar to females but with slightly slender and sharper tail, with paired and relatively strong spicules, and bursa peloderan. The population showed the following morphometrics: Females (*n* = 20) included L  =  493.97 ± 34.36 (458.45-556.38) μm, a = 25.98 ± 2.1 (22.24-28.72), b’ = 4.81 ± 0.35 (4.23-5.5), c = 15.76 ± 1.19 (13.46-17.59), c’ = 2.69 ± 0.29 (2.31-3.23), V = 57.59 ± 4.66 (50.00-66.11), V’ = 63.40 ± 3.91 (57.12-69.45), stylet length 16.02 ± 0.55 (15.36-17.29) μm, tail length 31.13 ± 2.16 (28.48-34.54) μm, M = 0.46 ± 0.03 (0.42-0.51), O = 0.16 ± 0.01 (0.14-0.17), MB = 0.57 ± 0.04 (0.52-0.63). Males (*n* = 20): L = 483.21 ± 29.78 (445.62-528.02) μm, a = 27.45 ± 2.1 (24.12-30.59), b’ = 4.80 ± 0.36 (4.24-5.34), c = 16.54 ± 0.98 (15.25-18.51), c’ = 2.29 ± 0.11 (2.07-2.51), stylet length 15.94 ± 0.47 (15.32-16.74) μm, tail length 29.42 ± 1.28 (27.37-31.53) μm, spicule length 18.20 ± 1.46 (15.65-21.00) μm, gubernaculum length 7.30 ± 0.68 (6.64-8.66) μm, M = 0.47 ± 0.02 (0.44-0.51), O = 0.16 ± 0.02 (0.13-0.18), MB = 0.61 ± 0.03 (0.54-0.67). The morphology of this population was in agreement with the original population of *Tylenchorhynchus zeae* identified in India (Sethi and Swarup, 1968). DNA was extracted from a random single nematodes (*n* = 10) using the Proteinase K method (Kumari and Subbotin, 2012), and the internal transcribed spacer1 (ITS1) region of rDNA and D2/D3 fragments of the 28 S rRNA were amplified with universal primers rDNA2 and rDNA1.58 s (Szalanski et al., 1997), D2A and D3B (Castillo et al., 2003), respectively. PCR products were sequenced by Tsingke Biological Technology (Beijing, China). The ITS1 sequence (MN757910, 721 bp) and D2/D3 sequence (MN757911, 802 bp) were submitted to GenBank and compared with published sequences by means of BLAST search in the database. The ITS1 sequence had a remarkably similarity of 99.00 to 99.05% with the *T. zeae* from Taiwan Province (EF519711) in China, and from Spain (KJ461597, KJ461598, KJ461599, KJ461600). The D2/D3 sequences exhibited 99.43 to 99.73% similarity with that of *T. zeae* from Iran (KM068058) and Spain (KJ461563, KJ461564, KJ461565, KJ461566). According with our morphological and molecular studies, this population was identified as *T. zeae*. The nematode was discovered from *Zea mays* in India (Sethi and Swarup, 1968), from cabbage, cauliflower and *Zea mays* in Taiwan Province, southeast China (Chen et al., 2007), from grapevine and olive in Spain (Handoo et al., 2014). To our knowledge, this is the first report of *T. zeae* in Gansu Province, northwest China, which expands the geographical distribution of the species.
